# Prognostic factors in canine appendicular osteosarcoma – a meta-analysis

**DOI:** 10.1186/1746-6148-8-56

**Published:** 2012-05-15

**Authors:** Ilse Boerman, Gayathri T Selvarajah, Mirjam Nielen, Jolle Kirpensteijn

**Affiliations:** 1 Department of Clinical Sciences of Companion Animals, Faculty of Veterinary Medicine, Utrecht University, Yalelaan 108, Utrecht, 3584, CM, The Netherlands; 2 Department of Veterinary Clinical Studies, Faculty of Veterinary Medicine, University Putra Malaysia, 43400 UPM, Serdang, Malaysia; 3 Department of Farm Animal Health, Faculty of Veterinary Medicine, Utrecht University, Yalelaan 107, Utrecht, 3584, CL, The Netherlands

**Keywords:** Bone tumor, Dog, Survival, Disease-free interval, Prognostic factors

## Abstract

**Background:**

Appendicular osteosarcoma is the most common malignant primary canine bone tumor. When treated by amputation or tumor removal alone, median survival times (MST) do not exceed 5 months, with the majority of dogs suffering from metastatic disease. This period can be extended with adequate local intervention and adjuvant chemotherapy, which has become common practice. Several prognostic factors have been reported in many different studies, e.g. age, breed, weight, sex, neuter status, location of tumor, serum alkaline phosphatase (SALP), bone alkaline phosphatase (BALP), infection, percentage of bone length affected, histological grade or histological subtype of tumor. Most of these factors are, however, only reported as confounding factors in larger studies. Insight in truly significant prognostic factors at time of diagnosis may contribute to tailoring adjuvant therapy for individual dogs suffering from osteosarcoma. The objective of this study was to systematically review the prognostic factors that are described for canine appendicular osteosarcoma and validate their scientific importance.

**Results:**

A literature review was performed on selected studies and eligible data were extracted. Meta-analyses were done for two of the three selected possible prognostic factors (SALP and location), looking at both survival time (ST) and disease free interval (DFI). The third factor (age) was studied in a qualitative manner. Both elevated SALP level and the (proximal) humerus as location of the primary tumor are significant negative prognostic factors for both ST and DFI in dogs with appendicular osteosarcoma. Increasing age was associated with shorter ST and DFI, however, was not statistically significant because information of this factor was available in only a limited number of papers.

**Conclusions:**

Elevated SALP and proximal humeral location are significant negative prognosticators for canine osteosarcoma.

## Background

Osteosarcoma (OS) is a malignant tumor of mesenchymal origin that produces osteoid. OS accounts for approximately 85 % of all primary canine bone tumors and is almost exclusively observed in large or giant breeds [1-5]. There is anecdotal evidence suggesting that males are more predisposed. The median age of onset of clinical signs ranges from 8 to 10 years [5,6], although it also occurs in younger dogs [7].

Dogs are often presented with a history of lameness or in some cases with a pathologic fracture of the affected bone. Predilection sites are the weight-bearing regions of the long bones (humerus, femur, radius, tibia and ulna) [8] with approximately 25 % of tumors arising in the axial skeleton including the flat bones of the skull, ribs, vertebrae, sternum, and pelvis [9,10]. OS is an aggressive and invasive neoplasm that causes local skeletal destruction and resulting in radiographic evidence of both osteoproductive and osteolytic lesions. They are highly metastatic, predominantly to the lungs with a lower frequency of spread to distant bones, regional lymph nodes [11] and other soft tissues [12,13]. A clinical diagnosis is made following assessment of case signalment and history and based on the radiographic appearance of the lesion. The definitive diagnosis is currently obtained by histological examination with tumor classification based on the formation of osteoid matrix with osteoblastic, fibroblastic, chondroblastic, telangiectic and combined subtypes [14,15]. There can be considerable variation in the histological appearance both between and within individual neoplasms, however, rendering histological parameters less reproducible between studies.

Dogs with OS that were treated by amputation alone have poor overall survival outcome: median overall survival times are typically less than five months, with the majority suffering from metastatic disease [5,16,17]. Over the years, advances in disease management, including ‘limb-sparing’ surgical and radioablative methods used only to selectively eradicate tumors located in the distal radius, ulna and tibia have been described [18,19]. Adjuvant therapy, such as multimodal chemotherapy regimes, treatment with bisphosphonates or immune modulators and palliative radiation, can also be provided and are reported to improve clinical or survival outcome [20-23]. Combination of therapy modalities and drugs may contribute significantly to survival statistics but randomized, double-blinded studies have not been performed routinely.

There are many well-documented prognostic indicators for canine OS, and the majority of these are similar to those reported from large retrospective studies on human OS, including tumor location, histologic grade, certain biomarkers, the use of chemotherapy, etc. [24-27]. The accurate segregation of canine patients into distinct prognostic subgroups based on such indicators is essential in the tailoring of appropriate treatment. However, the required information is not always completely available in the existing literature and studies report sometimes conflicting results. In this paper, we perform a meta-analysis to estimate the combined effect size over a number of studies of a selection of well-described prognosticators.

The aims of the current study were to systematically review the prognostic factors that are described for canine appendicular osteosarcoma and validate their scientific importance. After validation, a meta-analysis was performed on serum alkaline phosphatase (SALP), tumor location and age at diagnosis to study the association of these factors with survival time (ST) and disease free interval (DFI).

## Materials and methods

### Search strategy and quality assessment

A search was performed using Medline (PubMed) and Google Scholar search for all eligible studies performed between January 1970 and May 2011. The following search strategies were applied: [“canine”, “osteosarcoma” and “prognosis”] and [“canine”, “osteosarcoma” and “survival”]. Reports were selected and carefully reviewed that included canine stage <2B appendicular osteosarcoma cases [18]. Additionally, all manuscripts were evaluated for methodological quality according to a standardized questionnaire adapted from Bramer et al. (2009) including variables such as institute where the study was performed, year of publication, number of cases reported (at least 5), whether the study was randomized or not, study design, recorded survival end points, univariate (uva) or multivariate analysis (mva), use of control groups, listing of research period, completeness of follow up information, method of follow up examination, listing if cases were lost to follow up, definition of prognostic factors, listing of therapy modalities and listing of confounding factors. Two independent reviewers (JK, GS) performed study selection, assessment of methodological quality and data extraction. A third reviewer (IB) resolved disagreements, if necessary.

### Statistical analysis

Studies fulfilling one or two types of quality criteria were selected for meta-analysis. First, only randomized double-blind studies with multivariate data analysis were selected. After that, non-randomized, prospective studies were added to evaluate if additional significant factors could be determined. Studies with similar dog populations, outcome variables, prognostic factors, follow-up information, and adjustment for confounding variables were combined. Adjusted relative risks were pooled with a random effects model and assessed for statistical heterogeneity by Chi-square analysis. After establishment of heterogeneity, the source was determined by meta-analysis.

All meta-analyses were performed using commercial statistical software (Comprehensive meta-analysis V2, ©2006 Biostat, Inc., Englewood, NJ, USA), which provides a table with relative risk for each study and a forest plot. Both binary and continuous data were reported in the selected papers. It was impossible to combine both types of data in the same analysis; therefore for each variable separate sub analyses were performed on the effect measures hazard ratio (HR), median survival time (MST) and median disease free interval (MDFI). The groups compared for the variables SALP and location were as follows: elevated SALP versus SALP within reference range at time of diagnosis; (proximal) humerus compared to other locations in the appendicular skeleton. For age it was not possible to make two groups with the available data; factor data were analysed in a qualitative manner.

Some studies reported univariate data analysis while others reported multivariate data analysis, meaning that a studied factor was corrected for therapy or other confounding factors analysed. Where possible, data of multivariate analysis were used. Where necessary, a (set of) paper(s) was excluded to see if the overall effect changed. For all meta-analyses performed in this study the random-effects model was used, assuming that the true study effect varies across studies and the observed study effects reflect both this variation and random variation. Selected studies that did not present the data in sufficient detail were qualitatively summarized where necessary.

## Results

### Inclusion of papers

Through the searches, 821 papers were selected for review of which 55 met the criteria to be included in the study presented here (Additional file 1). No disagreements needed to be resolved by the third reviewer. Of the 55 studies, 16 were multicenter, and 39 monocenter, of which the majority came from Colorado State University (17), Utrecht University (5), University of Wisconsin-Madison and Tufts University (each 4). One study contained two independent studies and was split in two (Kurzman et al., 1995). One study was published in the 1970s, 5 in the 1980s, 14 in the 1990s and 35 in 2000–2011. 14 Studies were considered randomized, 27 studies were prospective, and 29 retrospective. Of all the studies, only 5 were placebo controlled. The number of cases varied from 11 to 303, with a mean of 61 ± 8. Outcome variables included survival time in 51, disease free interval in 32, metastasis free interval (MFI) in 13, and recurrence free interval (RFI) in 6 studies. Univariate analyses were performed in all studies varying from Kaplan Meier (KM) survival curves in 34, KM combined with (Cox) regression analysis in 20, and one study mentioned an ANOVA. Multivariate analysis was performed with a multivariate cox proportional hazard analysis in 18 and some other multivariate test in 7 reports. Historic control groups are common (n = 19) and some studies did not use a control group at all. The time of evaluation after the procedure and methodology varied too but most studies evaluated the dogs every 2–3 months using pulmonary radiography (n = 37); 13 studies did not include any information about follow up techniques. Most studies failed to include how long these dogs needed to be followed up (n = 39) or how many cases were lost to follow up (n = 33). An abundance of treatments were described, of which amputation combined with some kind of chemotherapy was most common.

### Significant factors

Many significant factors are described in the 55 papers selected, e.g. age, breed, weight, sex, neuter status, location of tumor, SALP, bone alkaline phosphatase (BAP), infection, percentage of bone length affected, histological grade or histological subtype of tumor. The effect of SALP level, location (proximal humerus) and age at diagnosis were reported most often in the 55 selected papers and these factors were subjected to meta-analyses.

Thirteen papers were included in the meta-analysis on SALP and seven papers in the meta-analysis on (proximal) humerus; some studies reported more than one variable. For age it turned out to be difficult to find sufficient studies with comparable selections of age categories to run meta-analysis on, therefore in the results a qualitative summation is given for all relevant age data found. Six papers reported the effect of age as confounding factor using HR, MST or MDFI, three papers did not give an estimate of the effect size, but only reported a p-value (Additional file 1: Table S 1).

### Serum alkaline phosphatase

#### Survival time

**Figure 1:**
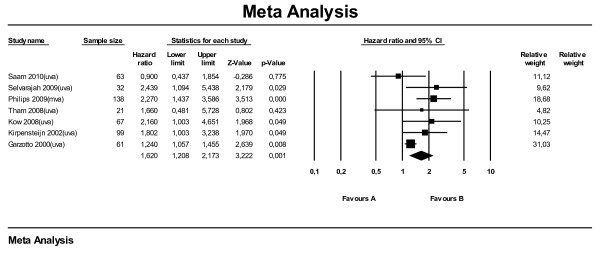
Meta-analysis of univariate and multivariate data sets for predictive value of serum alkaline phosphatase level on survival time using hazard ratios.

**Figure 2:**
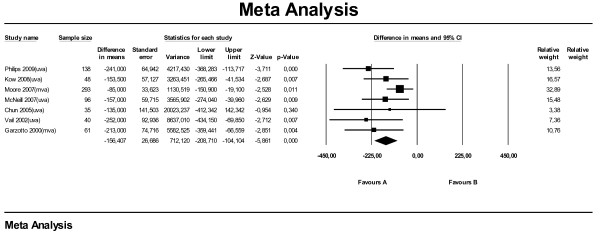
Meta-analysis of univariate and multivariate data sets for predictive value of serum alkaline phosphatase level on median survival time in days.

For the meta-analysis for SALP on survival time using hazard ratios, data from seven studies were available. Six of these studies used univariate analysis and one used multivariate analysis. All studies combined, the meta-analysis (Figure 1) on hazard ratios showed that dogs with an elevated SALP level at time of diagnosis have shorter survival times compared with dogs with a SALP level within reference range, with a hazard ratio of 1.62 (95 % CI: 1.21–2.17). Leaving the one study with the multivariate analysis (Phillips et al., 2009) out, the hazard ratio dropped to 1.44 (95 % CI: 1.11–1.87). The subanalysis with median survival times (also seven studies) showed that dogs with elevated SALP overall lived 156 days shorter (MST: -156) compared with dogs with SALP within reference range (95 % CI: -209 to −104) (Figure 2). When selecting only the studies with multivariate or univariate analysis the difference became −132 days (95 % CI: -252 to −11) and −186 days (95 % CI: -248 to −124), respectively.

#### Disease free interval

**Figure 3:**
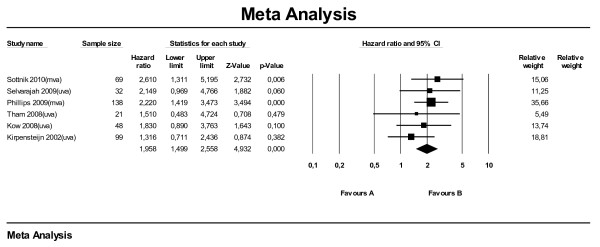
Meta-analysis of univariate and multivariate data sets for predictive value of serum alkaline phosphatase level on median disease free interval using hazard ratios.

**Figure 4:**
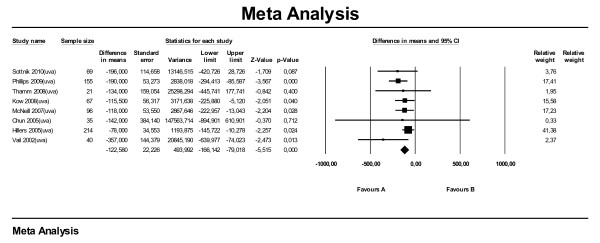
Meta-analysis of univariate and multivariate data sets for predictive value of serum alkaline phosphatase level on median disease free interval (in days).

For the meta-analysis on disease-free interval ten papers were used, of which the majority was also used for the meta-analysis on survival time. The effect direction of elevated SALP on DFI was comparable to that on ST. Figure 3 shows an overall HR of 1.96 (95 % CI: 1.50–2.56) for dogs with an elevated SALP level at time of diagnosis. The two multivariate analyses (Sottnik et al., 2010 and Phillips et al., 2009) together give a HR of 2.33 (95 % CI: 1.60–3.39) for elevated SALP level. Combining only the univariate analyses, the HR dropped to 1.64 (95 % CI: 1.12–2.40). The difference in MDFI (here only univariate analyses were available) was −123 days (95 % CI: -166 to −79) (Figure 4).

### Location - (proximal) humerus

#### Survival time

**Figure 5:**
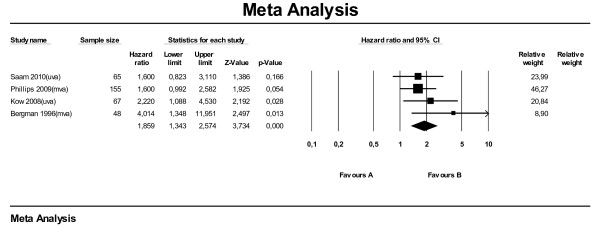
Meta-analysis of univariate and multivariate data sets for predictive value of location of tumor (LOC) on survival time using hazard ratios.

**Figure 6:**
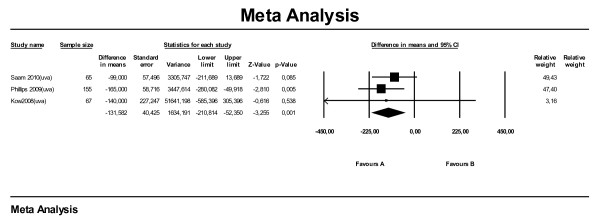
Meta-analysis of univariate data sets for predictive value of location of tumor (LOC) level on median survival time in days.

Four papers were used for the meta-analysis on ST for location. Of these papers, one reported the humerus as a whole as reference (Kow et al., 2008), the other three reported the proximal humerus in particular. Analysis on all studies combined (Figure 5) showed that dogs with the primary tumor located in their (proximal) humerus had shorter survival times than dogs with the primary tumor located elsewhere in their appendicular skeleton (HR 1.86; 95 % CI: 1.34–2.57). We also performed subset analyses on three subgroups (mva, uva and proximal humerus) and found no large differences in effect between the subgroups; estimates for HR varied between 1.82 and 2.21. The sub analysis on MST (Figure 6, all manuscripts reporting uva) showed that for (proximal) humerus the MST is 132 days shorter compared with other locations (95 % CI: -211 to −52). Approximately the same difference in MST (−131 days) applied for the sub analysis leaving out the proximal humerus.

#### Disease Free Interval

**Figure 7:**
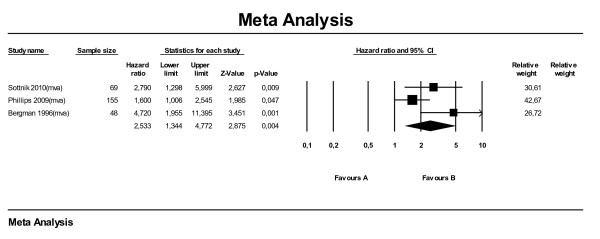
Meta-analysis of multivariate data sets for predictive value of location of tumor (LOC) on median disease free interval using hazard ratios.

**Figure 8:**
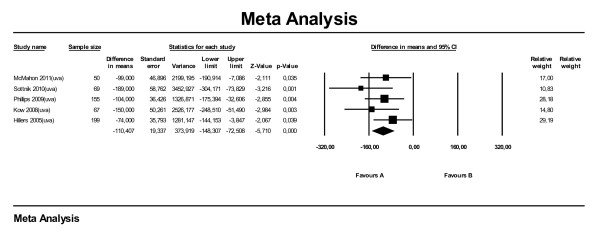
Meta-analysis of univariate data sets for predictive value of location of tumor (LOC) on median disease free interval (in days).

For the meta-analysis on DFI for location, data from six papers were used, three of which were also used for the analyses on ST. The effect of (proximal) humerus as location on DFI was comparable with the effect on ST. Dogs with primary tumors located in the (proximal) humerus had shorter DFIs compared with other locations (HR 2.53; 95 % CI: 1.34–4.77) (Figure 7). The effect of the proximal humerus in particular was comparable (HR 2.57; 95 % CI: 0.90–7.35). Performing the subanalysis on five papers reporting MDFI (only uva) the overall difference in MDFI was −110 days (95 % CI: -148 to −73) (Figure 8). Subset analyses on proximal humerus in particular and humerus as a whole showed MDFIs of respectively −91 and −167 days.

### Age

The papers analysing age as confounding factor all reported different age categories and used different effect outcomes. As it did not occur that more than two studies used both the same outcome type and the same age categories, it appeared impossible to run a meta-analysis on more than two studies. Therefore these data are summarized in a qualitative manner in Table 1, 2 and 3.

**Table 1:** Survival data on age of dogs with osteosarcoma coded as binary data

**Study (UVA/MVA)**	**N**	**Studied age categories (years)**	**MST (days)**	**HR (95 % CI)**	**p-value**
Saam 2010 (UVA)	12	<=5	1414	0.49 (0.3-1.1)	0.08
	53	>5	265		
Phillips 2009 (UVA)	23	<5	1263		0.034
	132	>5	293		
Miller 2009 (UVA)	3	<6		(reference)	0.89
	73	6-10		1.09 (0.21-5.68)	
	7	>10		1.14 (0.28-4.71)	
Liptak 2006 (MVA)	20	Older*		2.06 (1.03-4.14)	0.042
Kent 2004 (UVA)	32	<mean age**			0.156
Spodnick 1992 (MVA)	162	7-10*** vs.			<0.01
		<7 and >10			

UVA = univariate analysis; MVA = multivariate analysis; N = number; MST = median survival time; HR = hazard ration; reference = the reference group with which the others are compared.

*MST for older dogs was significantly decreased (median age 8.3 years, range 4.5-11 years).

**Only median age was mentioned in article (8.8 years, range 2.2-16.5 years)

***Longer survival time.

* MST for older dogs was significantly decreased (median age 8.3 years, range 4.5-11).

** Median age 8.8 years, range 2.2–16.5.

*** Longer ST.

**Table 2:** Survival data of dogs with osteosarcoma coded as continuous data

**Study (UVA/MVA)**	**N**	**Continuous variable**	**MST (days)**	**HR (95 % CI)**	**p-value**
Selvarajah 2009 (UVA)	32	Overall		1.137 (0.96-1.35)	0.147
Selvarajah 2009 (MVA)	32	Overall	204	1.18 (0.92-1.52)	0.202
Moore 2007 (MVA)	303	Increasing age	244*		0.004

* MST was recalculated from months (8) to days by multiplying with 30.5.

#### Survival time

The influence of age on ST reported in the eight papers used is shown in Table 1 and 2. According to Saam et al. (2011) and Phillips et al. (2009), dogs under the age of 5 showed longer MSTs compared with dogs over the age of 5. This was comparable to the study of Liptak, et al. (2006), which reported that older dogs had a significantly shorter MST. In the study of Miller et al. (2009), dogs older than 6 years showed a slightly increased HR (but with insignificant p-value) compared with younger dogs. The study of Kent et al. (2004) reported that the age under the mean age in that study was not prognostic for survival. According to Spodnick et al. (1992) dogs in the age between 7 and 10 years showed a significantly longer MST than dogs under 7 or over 10 years old. Concerning the continuous survival data; in the paper of Selvarajah et al. (2009) age overall did not significantly influence ST. Moore et al. (2007) reported that dogs have significantly worse survival rates as age increases. Overall, most studies performed in the last decennium showed worse survival rates for dogs with increasing age compared with younger dogs.

#### Disease free interval

Contradictory data were observed for DFI (Table 3). According to the study of Sottnik et al. (2010), dogs older than 8 years showed significantly longer MDFIs compared with younger dogs. Phillips et al. (2009) on the other hand reported that dogs older than 5 showed a significantly increased HR for MDFI and significantly shorter MDFIs compared with younger dogs. Miller et al. (2009) also reported an increased HR for dogs older than 6 years, however, not significantly. Last, in the study of Kent et al. (2004) age under the mean age was prognostic for DFI.

**Table 3:** Disease free interval data on age of dogs with osteosarcoma coded as binary data

**Study (UVA/MVA)**	**N**	**Studied age categories (years)**	**Median DFI (days)**	**HR (95% CI)**	***P*-value**
Sottnik 2010 (UVA)	39	<=8	204		0.188
	30	>8	345	0.59	
Sottnik 2010 (MVA)	69	>8 vs. <=8		0.42 (0.21-0.85)	**0.016**
Phillips 2009 (UVA)	23	<5	1035		**0.022**
	132	>5	241		
Phillips 2009 (MVA)	155	>5 vs. <5		2.1 (1.04-4.25)	**0.038**
Miller 2009 (UVA)	3	<6		(reference)	0.54
	73	6-10		1.59 (0.33-7.71)	
	7	>10		1.21 (0.3-4.96)	
Kent 2004 (UVA)	32	<mean			0.049*

UVA = univariate analysis; MVA = multivariate analysis; N = number; DFI = disease free interval; HR = hazard ration; reference = the reference group with which the others are compared.

* DFI in this paper was stated as ‘progression free survival’.

## Discussion

This is the first paper published in veterinary literature that compares outcomes in companion animal osteosarcoma using meta-analysis confirming the significance of three prognostic variables, including serum alkaline phosphatase, location and age in this devastating tumor.

### Serum ALP is a strong prognosticator

SALP is most likely the strongest prognostic indicator for ST and DFI of canine OS and confirmed expectations in the veterinary literature. Bone alkaline phosphatase is a bone turnover marker measured in serum. It reflects the increased turnover associated with bone destruction or aging and various conditions affecting bone metabolism [28], such as osteosarcoma. The prognostic significance of serum total alkaline phosphatase activity for human beings with osteosarcoma has been recognized for over 30 years [29]. In most veterinary studies, the total SALP is measured. Total SALP is easily quantifiable and a routine diagnostic test in most veterinary laboratories. Total SALP, however, consists of several other isoenzymes, of which those derived from liver (LALP) and bone (BALP) represent the majority in normal dogs. A corticosteroid-induced isoenzyme (CALP) may be present in dogs with hyperadrenocorticism and dogs receiving exogenous corticosteroids [30]. The prognostic value of total SALP for human osteosarcoma is limited by its lack of specificity for tumor tissue [31,32]. The enzyme’s activity may be increased by exogenous corticosteroid administration or hyperadrenocorticism, which may cause increases in CALP, or by cholestasis which may cause increases in LALP [31]. Measuring the total SALP might therefore not always give reliable outcomes. Measuring the bone ALP (BALP) seems more logical, since in dogs with osteosarcoma, BALP is expected and proven to be elevated and more discriminant than the other isoenzymes [33]. Serum BALP activity was a direct reflection of osteoblastic activity [34] in people. Additionally, the reference range for SALP in dogs differs among various age groups [33,35] and breeds [36], which could be another factor that may influence study results. The overall meta-analysis done in this study for median ST showed a stronger significant effect on HR than the analysis on only MVA or UVA studies. This could be caused by confounding variables that may be associated with SALP, including breed variability, underlying disease processes and differences in detection methods.

### Proximal humeral location is associated with poor survival

The most common human OS sites are the femur (42 %), tibia (19 %), and humerus (10 %) [37]. The proximal humerus, despite the fact that it is not the most common site, was associated with poorer (metastasis-free) survival [38,39] in man. In this study, the (proximal) humerus was also shown to be the location with a worse prognostic outcome for canine OS. One of the reasons is the fact that tumors in this location may be observed at a later stage compared to other locations that are more clinically obvious.

The location with the best prognosis may also be of clinical relevance for both canine and human OS, however for canine OS this information was often not available. In human OS, survival of patients with primary malignant bone tumor of distal lower extremity seems to be better than that of other sites [40]. This is comparable to canine OS: in several canine studies, the radius is cited as the location with the best prognosis for ST and DFI [2,41-43].

### Age may be prognostic in canine osteosarcoma

In human OS, a primary osteosarcoma in older patients showed a poorer prognosis [38,44]. A predisposing factor in middle-aged to elderly people is Paget’s disease, however. One of the most serious complications of Paget’s disease is a significant increase in the incidence of osteosarcoma [45]. Despite of the limited amount of canine OS data available for age analyses, it seemed that increasing age is an important prognostic factor for dogs with appendicular OS. Survival time may not be the most sensitive variable, however, because it can be confounded by various other medical problems that arise in older dogs with malignant bone cancer. Additional studies comparing age categories need to be conducted using more elaborate meta-analyses.

### The role of adjuvant chemotherapy

Over the years, various compounds have been used in adjuvant chemotherapy protocols against canine OSA including cisplatin, carboplatin and doxorubicin. These have been used in single and in multi-agent regimes and at varying dosage and treatment interval and an obvious survival advantage of dogs receiving chemotherapy was present compared to (historic) controls [46-49]. No obvious differences in survival were observed when these treatments were compared with pre- or post-operative chemotherapy [50] and no differences in the DFI was reported for dogs treated using single- or multi-agent chemotherapeutic regimes. The prolonged, intense use of chemotherapy is often not a valid option due to adverse side effects compromising any clinical benefits and decreasing client compliance [51]. To date, even aggressive adjunctive therapy has proven ineffective in restricting all growth of metastases. Additionally, a small number of cases of canine OS that do not receive adjuvant chemotherapy do not succumb to metastatic disease once the primary tumor has been removed [52]. This finding suggests that genetic composition of both the host and tumor may be contributing to differences in the metastatic potential. In human OS, the prognosis has increased from 20 % 5-year survival in 1970 to 60 % nowadays because of the various chemotherapeutic protocols [53]. The histological response to preoperative chemotherapy was an important clinical predictor of the result of operative treatment of human osteosarcoma and was similarly important in one study by Powers et al [54]. This indicator should be used to identify patients who are at high risk for metastasis; as such patients may be candidates for more intensive or novel therapy protocols [42]. This important prognostic factor in human OS may also be a very interesting factor to further investigate in the canine OS but very few studies have reported evaluation of this variable making meta-analysis currently impossible. Meta-analyses for this factor and for different (types of) chemotherapy may be a valuable next step to see which therapies give the most optimal results for survival in canine appendicular osteosarcoma.

### New insights, limitations and recommendations for prognostic studies in canine OSA

Often scientific papers only report data when they are found significant. Non-significant results are many times only cited as ‘non significant’, yet the statistical data are left out. To be able to perform a meta-analysis taking into account all relevant data both significant and non-significant outcomes are essential. Leaving out data may result in an inadequate outcome. Unfortunately, not all available papers could be used for the meta-analyses in this study, since results were not always reported in sufficient detail whereby essential data were missing (e.g. sample size, confidence interval, HR or p-value).

For future reference, data should be coded in a manner that allows comparison of various studies with comparable objectives. For instance, we observed that in some studies data were coded as continuous variables, in other studies they are coded as binary data. Therefore, the estimates of the effect size are difficult to compare. When specific factors are coded >1, one would expect them to be a negative prognostic factor. Once coded <0 it would be a protective factor. This was sometimes mixed up. For example, in the study of Phillips (2009) elevated SALP was cited as ‘negative prognostic factor’, yet the corresponding HR was 0.44. Since on the other hand following uva analysis both ST and MST for dogs with elevated SALP was shorter than for dogs with SALP within reference range, it seemed that the HR should be 1/0.44 [55]. The paper of Sottnik, et al (2010) was not completely clear whether the HR of 0.59 corresponded to the age category ≤8 (which was the correspondent category in the study or otherwise not stated clearly in the relevant Table) or ≥8 years [56]. For the meta-analysis in the current study the HR was linked to the category ≥8 years, since in the mva analysis the same category also showed a HR <1 (0.42) and the MDFIs were also longer for that specific category. Yet the range of the DFI (uva) in the category ≤8 was extremely large which could mean that the data are not completely reliable. All in all, the systematic coding of risk factors in a consequent way would be extremely helpful for future analyses.

Although many studies are performed, on account of above-mentioned reasons it remains difficult or even impossible to compare these individual studies and significantly prove that possible prognostic factors are really prognostic. Only a few studies could be used for the meta-analyses and of these studies it needs to be said that we stretched to the limit the fact that the studies itself were comparable. This is a common and unavoidable fact for diseases that are relatively infrequent, such as canine OS. Researchers should therefore be stimulated to work together in the OS field, which is not only interesting from a research point of view but also in in the One Health approach of this comparable disease between man and dog.

## Conclusions

Both elevated SALP level and the (proximal) humerus as location of the primary tumor are significant negative prognostic factors for both ST and DFI in dogs with appendicular osteosarcoma. Increasing age was associated with shorter ST and DFI, however, was not statistically significant because information of this factor was available in only a limited number of papers. Multicenter, well-designed research efforts are necessary to confound the message that should be relayed to clients and patients to allow them to make an evidence-based decision in the treatment of their animal or child with this type of malignant bone tumor. Multicenter studies are only possible when researchers use the same variable definitions and show all relevant results.

## Abbreviations

OS: Osteosarcoma; SALP: Serum alkaline phosphatase; BAP: Bone alkaline phosphatase; (M)ST: (Median) Survival Time; (M)DFI: (Median) Disease Free Interval; MFI: Metastasis free interval; RFI: Recurrence free interval; HR: Hazard ratio; UVA: Univariate; MVA: Multivariate.

## Competing interests

The author(s) declare that they have no competing interests.

## Authors’ contributions

IB carried out the manuscript search, databasing the various studies, performing the meta-analysis and drafted the manuscript. GTS helped with the manuscript search and reviewed the selected candidate manuscripts. MN supervised the meta-analysis. JK conceived the idea of the study, and participated in its design and coordination and helped to draft the manuscript. All authors read and approved the final manuscript.

## Supplementary Material

Additional file 1 ** Papers (55) that met the criteria for inclusion in this study [57-107]. Table S1.** Studies selected for meta-analysis. Click here for file

## References

[B1] Cooley DM Waters DJ Skeletal neoplasms of small dogs: a retrospective study and literature review J Am Anim Hosp Assoc 1997 33 11 23 8974021 10.5326/15473317-33-1-11

[B2] McNeill CJ Overley B Shofer FS Kent MS Clifford CA Samluk M Haney S Van Winkle TJ Sorenmo KU Characterization of the biological behaviour of appendicular osteosarcoma in Rottweilers and a comparison with other breeds: a review of 258 dogs Vet Comp Oncol 2007 5 90 98 10.1111/j.1476-5829.2006.00116.x 19754792

[B3] Norrdin RW Powers BE Torgersen JL Smith RE Withrow SJ Characterization of osteosarcoma cells from two sibling large-breed dogs Am J Vet Res 1989 50 1971 1975 2619126

[B4] Ru G Terracini B Glickman LT Host related risk factors for canine osteosarcoma Vet J 1998 156 31 39 10.1016/S1090-0233(98)80059-2 9691849

[B5] Spodnick GJ Berg J Rand WM Schelling SH Couto G Harvey HJ Henderson RA MacEwen G Mauldin N McCaw DL Prognosis for dogs with appendicular osteosarcoma treated by amputation alone: 162 cases (1978–1988) J Am Vet Med Assoc 1992 200 995 999 1577656

[B6] Boston SE Ehrhart NP Dernell WS Lafferty M Withrow SJ Evaluation of survival time in dogs with stage III osteosarcoma that undergo treatment: 90 cases (1985–2004) J Am Vet Med Assoc 2006 228 1905 1908 10.2460/javma.228.12.1905 16784383

[B7] Evans LB Osteosarcoma in a young Great Dane dog J S Afr Vet Assoc 1983 54 271 273 6583421

[B8] Liptak JM Dernell WS Straw RC Rizzo SA Lafferty MH Withrow SJ Proximal radial and distal humeral osteosarcoma in 12 dogs J Am Anim Hosp Assoc 2004 40 461 467 15533966 10.5326/0400461

[B9] Dickerson ME Page RL LaDue TA Hauck ML Thrall DE Stebbins ME Price GS Retrospective analysis of axial skeleton osteosarcoma in 22 large-breed dogs J Vet Intern Med 2001 15 120 124 10.1111/j.1939-1676.2001.tb01242.x 11300594

[B10] Hammer AS Weeren FR Weisbrode SE Padgett SL Prognostic factors in dogs with osteosarcomas of the flat or irregular bones J Am Anim Hosp Assoc 1995 31 321 326 7552665 10.5326/15473317-31-4-321

[B11] Hillers KR Dernell WS Lafferty MH Withrow SJ Lana SE Incidence and prognostic importance of lymph node metastases in dogs with appendicular osteosarcoma: 228 cases (1986–2003) J Am Vet Med Assoc 2005 226 1364 1367 10.2460/javma.2005.226.1364 15844430

[B12] Gorman E Barger AM Wypij JM Pinkerton ME Cutaneous metastasis of primary appendicular osteosarcoma in a dog Vet Clin Pathol 2006 35 358 361 10.1111/j.1939-165X.2006.tb00149.x 16967427

[B13] Peremans K Otte A Verschooten F Van Bree H Dierckx R Soft tissue metastasis of an osteosarcoma of the humerus in a four-legged patient Eur J Nucl Med Mol Imaging 2003 30 188 10.1007/s00259-002-1041-9 12483426

[B14] Kirpensteijn J Kik M Rutteman GR Teske E Prognostic significance of a new histologic grading system for canine osteosarcoma Vet Pathol 2002 39 240 246 10.1354/vp.39-2-240 12009062

[B15] Loukopoulos P Robinson WF Clinicopathological relevance of tumour grading in canine osteosarcoma J Comp Pathol 2007 136 65 73 10.1016/j.jcpa.2006.11.005 17270206

[B16] Brodey RS Abt DA Results of surgical treatment in 65 dogs with osteosarcoma J Am Vet Med Assoc 1976 168 1032 1035 1064592

[B17] Straw RC Withrow SJ Richter SL Powers BE Klein MK Postorino NC LaRue SM Ogilvie GK Vail DM Morrison WB Amputation and cisplatin for treatment of canine osteosarcoma J Vet Intern Med 1991 5 205 210 10.1111/j.1939-1676.1991.tb00950.x 1941754

[B18] Boston SE Duerr F Bacon N Larue S Ehrhart EJ Withrow S Intraoperative radiation for limb sparing of the distal aspect of the radius without transcarpal plating in five dogs Vet Surg 2007 36 314 323 10.1111/j.1532-950X.2007.00272.x 17547594

[B19] Straw RC Withrow SJ Limb-sparing surgery versus amputation for dogs with bone tumors Vet Clin North Am Small Anim Pract 1996 26 135 143 8825572 10.1016/s0195-5616(96)50012-4

[B20] Dow S Elmslie R Kurzman I MacEwen G Pericle F Liggitt D Phase I study of liposome-DNA complexes encoding the interleukin-2 gene in dogs with osteosarcoma lung metastases Hum Gene Ther 2005 16 937 946 10.1089/hum.2005.16.937 16076252

[B21] Fan TM Charney SC de Lorimier LP Garrett LD Griffon DJ Gordon-Evans WJ Wypij JM Double-blind placebo-controlled trial of adjuvant pamidronate with palliative radiotherapy and intravenous doxorubicin for canine appendicular osteosarcoma bone pain J Vet Intern Med 2009 23 152 160 10.1111/j.1939-1676.2008.0221.x 19175734

[B22] Tomlin JL Sturgeon C Pead MJ Muir P Use of the bisphosphonate drug alendronate for palliative management of osteosarcoma in two dogs Vet Rec 2000 147 129 132 10.1136/vr.147.5.129 10958534

[B23] Walter CU Dernell WS LaRue SM Lana SE Lafferty MH LaDue TA Withrow SJ Curative-intent radiation therapy as a treatment modality for appendicular and axial osteosarcoma: a preliminary retrospective evaluation of 14 dogs with the disease Vet Comp Oncol 2005 3 1 7 10.1111/j.1476-5810.2005.00062.x 19379208

[B24] Bramer JA van Linge JH Grimer RJ Scholten RJ Prognostic factors in localized extremity osteosarcoma: a systematic review Eur J Surg Oncol 2009 35 1030 1036 10.1016/j.ejso.2009.01.011 19232880

[B25] Kim MS Lee SY Cho WH Song WS Koh JS Lee JA Yoo JY Jeon DG Tumor necrosis rate adjusted by tumor volume change is a better predictor of survival of localized osteosarcoma patients Ann Surg Oncol 2008 15 906 914 10.1245/s10434-007-9779-8 18163171

[B26] Owen LN Comparative aspects of bone tumours in man and dog Proc R Soc Med 1967 60 1309 1310 5235121 10.1177/003591576706001225 PMC1901463

[B27] Pakos EE Nearchou AD Grimer RJ Koumoullis HD Abudu A Bramer JA Jeys LM Franchi A Scoccianti G Campanacci D Capanna R Aparicio J Tabone MD Holzer G Abdolvahab F Funovics P Dominkus M Ilhan I Berrak SG Patino-Garcia A Sierrasesumaga L San-Julian M Garraus M Petrilli AS Filho RJ Macedo CR Alves MT Seiwerth S Nagarajan R Cripe TP Ioannidis JP Prognostic factors and outcomes for osteosarcoma: an international collaboration Eur J Cancer 2009 45 2367 2375 10.1016/j.ejca.2009.03.005 19349163

[B28] Jenkins DK Bone alkaline phosphatase, a serum bone turnover assay: useful in managing postmenopausal women receiving therapy to prevent or treat osteoporosis 2010 Quidel Corporation, 217A US 9/01

[B29] McKenna RJ Schwinn CP Soong KY Higinbotham NL Osteogenic Sarcoma Arising in Paget's Disease Cancer 1964 17 42 66 10.1002/1097-0142(196401)17:1<42::AID-CNCR2820170108>3.0.CO;2-U 14102071

[B30] Kramer JW HWE Clinical enzymology Clinical Biochemistry of Domestic Animals 1997 5 Academic Press, San Diego, CA 303 325

[B31] Levine AM Rosenberg SA Alkaline phosphatase levels in osteosarcoma tissue are related to prognosis Cancer 1979 44 2291 2293 10.1002/1097-0142(197912)44:6<2291::AID-CNCR2820440643>3.0.CO;2-S 292511

[B32] Liu PP Leung KS Kumta SM Lee KM Fung KP Bone-specific alkaline phosphatase in plasma as tumour marker for osteosarcoma Oncology 1996 53 275 280 10.1159/000227573 8692530

[B33] Ehrhart N Dernell WS Hoffmann WE Weigel RM Powers BE Withrow SJ Prognostic importance of alkaline phosphatase activity in serum from dogs with appendicular osteosarcoma: 75 cases (1990–1996) J Am Vet Med Assoc 1998 213 1002 1006 9776998

[B34] Leung KS Fung KP Sher AH Li CK Lee KM Plasma bone-specific alkaline phosphatase as an indicator of osteoblastic activity J Bone Joint Surg Br 1993 75 288 292 8444951 10.1302/0301-620X.75B2.8444951

[B35] Allen LC Allen MJ Breur GJ Hoffmann WE Richardson DC A comparison of two techniques for the determination of serum bone-specific alkaline phosphatase activity in dogs Res Vet Sci 2000 68 231 235 10.1053/rvsc.1999.0369 10877968

[B36] Nestor DD Holan KM Johnson CA Schall W Kaneene JB Serum alkaline phosphatase activity in Scottish Terriers versus dogs of other breeds J Am Vet Med Assoc 2006 228 222 224 10.2460/javma.228.2.222 16426191

[B37] Jerome TJ Varghese M Sankaran B Thomas S Thirumagal SK Tibial chondroblastic osteosarcoma–case report Foot Ankle Surg 2009 15 33 39 10.1016/j.fas.2008.04.008 19218063

[B38] Cho WH Song WS Jeon DG Kong CB Kim MS Lee JA Yoo JY Kim JD Lee SY Differential presentations, clinical courses, and survivals of osteosarcomas of the proximal humerus over other extremity locations Ann Surg Oncol 2010 17 702 708 10.1245/s10434-009-0825-6 19921336

[B39] Song WS Kong CB Jeon DG Cho WH Kim MS Lee JA Yoo JY Kim JD Lee SY Prognosis of extremity osteosarcoma in patients aged 40–60 years: a cohort/case controlled study at a single institute Eur J Surg Oncol 2010 36 483 488 10.1016/j.ejso.2010.03.006 20363585

[B40] Yan TQ Guo W Yang RL Sun X Qu HY The survival and functional outcome of primary bone sarcomas in distal lower extremity Zhonghua Wai Ke Za Zhi 2010 48 1550 1555 21176669

[B41] Bacon NJ Ehrhart NP Dernell WS Lafferty M Withrow SJ Use of alternating administration of carboplatin and doxorubicin in dogs with microscopic metastases after amputation for appendicular osteosarcoma: 50 cases (1999–2006) J Am Vet Med Assoc 2008 232 1504 1510 10.2460/javma.232.10.1504 18479240

[B42] Sami SH Rafati AH Hodjat P Tissue necrosis after chemotherapy in osteosarcoma as the important prognostic factor Saudi Med J 2008 29 1124 1129 18690304

[B43] Saam DE Liptak JM Stalker MJ Chun R Predictors of outcome in dogs treated with adjuvant carboplatin for appendicular osteosarcoma: 65 cases (1996–2006) J Am Vet Med Assoc 2011 238 195 206 10.2460/javma.238.2.195 21235373

[B44] Jeon DG Lee SY Cho WH Song WS Park JH Primary osteosarcoma in patients older than 40 years of age J Korean Med Sci 2006 21 715 718 10.3346/jkms.2006.21.4.715 16891818 PMC2729896

[B45] Hansen MF Nellissery MJ Bhatia P Common mechanisms of osteosarcoma and Paget's disease J Bone Miner Res 1999 14 39 44 10510212 10.1002/jbmr.5650140209

[B46] Bailey D Erb H Williams L Ruslander D Hauck M Carboplatin and doxorubicin combination chemotherapy for the treatment of appendicular osteosarcoma in the dog J Vet Intern Med 2003 17 199 205 10.1111/j.1939-1676.2003.tb02434.x 12683621

[B47] Berg J Weinstein MJ Springfield DS Rand WM Results of surgery and doxorubicin chemotherapy in dogs with osteosarcoma J Am Vet Med Assoc 1995 206 1555 1560 7775232

[B48] Bergman PJ MacEwen EG Kurzman ID Henry CJ Hammer AS Knapp DW Hale A Kruth SA Klein MK Klausner J Norris AM McCaw D Straw RC Withrow SJ Amputation and carboplatin for treatment of dogs with osteosarcoma: 48 cases (1991 to 1993) J Vet Intern Med 1996 10 76 81 10.1111/j.1939-1676.1996.tb02031.x 8683484

[B49] Chun R Kurzman ID Couto CG Klausner J Henry C MacEwen EG Cisplatin and doxorubicin combination chemotherapy for the treatment of canine osteosarcoma: a pilot study J Vet Intern Med 2000 14 495 498 10.1111/j.1939-1676.2000.tb02265.x 11012111

[B50] Berg J Gebhardt MC Rand WM Effect of timing of postoperative chemotherapy on survival of dogs with osteosarcoma Cancer 1997 79 1343 1350 10.1002/(SICI)1097-0142(19970401)79:7<1343::AID-CNCR11>3.0.CO;2-# 9083156

[B51] Barabas K Milner R Lurie D Adin C Cisplatin: a review of toxicities and therapeutic applications Vet Comp Oncol 2008 6 1 18 10.1111/j.1476-5829.2007.00142.x 19178659

[B52] Selvarajah GT Kirpensteijn J van Wolferen ME Rao NA Fieten H Mol JA Gene expression profiling of canine osteosarcoma reveals genes associated with short and long survival times Mol Cancer 2009 8 72 10.1186/1476-4598-8-72 19735553 PMC2746177

[B53] Cho Y Jung GH Chung SH Kim JY Choi Y Kim JD Long-term survivals of stage IIb osteosarcoma: a 20-year experience in a single institution Clin Orthop Surg 2011 3 48 54 10.4055/cios.2011.3.1.48 21369478 PMC3042169

[B54] Powers BE Withrow SJ Thrall DE Straw RC LaRue SM Page RL Gillette EL Percent tumor necrosis as a predictor of treatment response in canine osteosarcoma Cancer 1991 67 126 134 10.1002/1097-0142(19910101)67:1<126::AID-CNCR2820670123>3.0.CO;2-7 1985708

[B55] Phillips B Powers BE Dernell WS Straw RC Khanna C Hogge GS Vail DM Use of single-agent carboplatin as adjuvant or neoadjuvant therapy in conjunction with amputation for appendicular osteosarcoma in dogs J Am Anim Hosp Assoc 2009 45 33 38 19122062 10.5326/0450033

[B56] Sottnik JL Rao S Lafferty MH Thamm DH Morley PS Withrow SJ Dow SW Association of blood monocyte and lymphocyte count and disease-free interval in dogs with osteosarcoma J Vet Intern Med 2010 24 1439 1444 10.1111/j.1939-1676.2010.0591.x 20840314

[B57] Bacon NJ Erhart NP Dernell WS Use of alternating administration of carboplatin and doxorubicin in dogs with microscopic metastases after amputation for appendicular osteosarcoma: 50 cases (1999-2006) J Am Vet Med Assoc 2008 May 15 232 10 1504 10 10.2460/javma.232.10.1504 18479240

[B58] Bech-Nielsen S Brodey RS Fidler IJ The effect of BCG on in vitro immune reactivity and clinical course in dogs treated surgically for osteosarcoma Eur J Cancer 1977 Jan 13 33 41 321227 10.1016/0014-2964(77)90227-4

[B59] Berg J Weinstein MJ Schelling SH Rand WM Treatment of dogs with osteosarcoma by administration of cisplatin after amputation or limb-sparing surgery: 22 cases (1987-1990) J Am Vet Med Assoc 1992 Jun 15 200 12 2005 8 1639715

[B60] Bergman PJ MacEwen EG Kurzman ID Amputation and carboplatin for treatment of dogs with osteosarcoma: 48 cases (1991 to 1993) J Vet Intern Med 1996 Mar-Apr 10 2 76 81 10.1111/j.1939-1676.1996.tb02031.x 8683484

[B61] Biller BJ Guth A Burton JH Dow SW Decreased ratio of CD81 T cells to regulatory T cells associated with decreased survival in dogs with osteosarcoma J Vet Intern Med 2010 24 1118 1123 10.1111/j.1939-1676.2010.0557.x 20666983 PMC3557512

[B62] Chun R Garrett LD Henry C Toxicity and efficacy of cisplatin and doxorubicin combination chemotherapy for the treatment of canine osteosarcoma J Am Anim Hosp Assoc 2005 Nov-Dec 41 6 382 7 16267062 10.5326/0410382

[B63] DiResta GR Aiken SW Brown HK Use of an artificial lymphatic system during carboplatin infusion to improve canine osteosarcoma blood flow and clinical response Ann Surg Oncol 2007 Aug 14 8 2411 21 10.1245/s10434-007-9373-0 17503157

[B64] Ehrhart N Dernell WS Hoffmann WE Prognostic importance of alkaline phosphatase activity in serum from dogs with appendicular osteosarcoma: 75 cases (1990-1996) J Am Vet Med Assoc 1998 Oct 1 213 7 1002 6 9776998

[B65] Fieten H Spee B Ijzer J Expression of hepatocyte growth factor and the proto-oncogenic receptor c-Met in canine osteosarcoma Vet Pathol 2009 Sep 46 5 869 77 10.1354/vp.08-VP-0155-F-FL 19429984

[B66] Garzotto CK Berg J Hoffmann WE Rand WM Prognostic significance of serum alkaline phosphatase activity in canine appendicular osteosarcoma J Vet Intern Med 2000 14 587 592 10.1111/j.1939-1676.2000.tb02281.x 11110379

[B67] Hahn KA Legendre AM Talbott JR The frequency of micronuclei in lymphocytes of dogs with osteosarcoma: a predictive variable for tumor response during cisplatin chemotherapy Cancer Epidemiol Biomarkers Prev 1996 Aug 5 8 653 6 8824369

[B68] Hahn KA Legendre AM Schuller HM Amputation and dexniguldipine as treatment for canine appendicular osteosarcoma J Cancer Res Clin Oncol 1997 123 1 34 8 10.1007/BF01212612 8996538 PMC12200756

[B69] Hillers KR Dernell WS Lafferty MH Incidence and prognostic importance of lymph node metastases in dogs with appendicular osteosarcoma: 228 cases (1986-2003) J Am Vet Med Assoc 2005 Apr 15 226 8 1364 7 10.2460/javma.2005.226.1364 15844430

[B70] Kent MS Strom A London CA Alternating carboplatin and doxorubicin as adjunctive chemotherapy to amputation or limb-sparing surgery in the treatment of appendicular osteosarcoma in dogs J Vet Intern Med 2004 18 540 544 10.1111/j.1939-1676.2004.tb02582.x 15320595

[B71] Khanna C Prehn J Hayden D A randomized controlled trial of octreotide pamoate long-acting release and carboplatin versus carboplatin alone in dogs with naturally occurring osteosarcoma: evaluation of insulin-like growth factor suppression and chemotherapy Clin Cancer Res 2002 Jul 8 7 2406 12 12114446

[B72] Khanna C Wan X Bose S The membrane-cytoskeleton linker ezrin is necessary for osteosarcoma metastasis Nat Med 2004 Feb 10 2 182 6 10.1038/nm982 14704791

[B73] Kirpensteijn J Kik M Rutteman GR Teske E Prognostic significance of a new histologic grading system for canine osteosarcoma Vet Pathol 2002 Mar 39 2 240 6 10.1354/vp.39-2-240 12009062

[B74] Kirpensteijn J Timmermans-Spran EP van Garderen E Growth hormone gene expression in canine normal growth plates and spontaneous osteosarcoma Mol Cell Endocrinol 2002 Nov 29 197 1–2 179 85 12431811 10.1016/s0303-7207(02)00269-1

[B75] Kirpensteijn J Kik M Teske E Rutteman GR TP53 gene mutations in canine osteosarcoma Vet Surg 2008 Jul 37 5 454 60 10.1111/j.1532-950X.2008.00407.x 18986312

[B76] Kow K Thamm DH Terry J Impact of telomerase status on canine osteosarcoma patients J Vet Intern Med 2008 Nov-Dec 22 6 1366 72 10.1111/j.1939-1676.2008.0175.x 18761602

[B77] Kuntz CA Asselin TL Dernell WS Limb salvage surgery for osteosarcoma of the proximal humerus: outcome in 17 dogs Vet Surg 1998 Sep-Oct 27 5 417 22 10.1111/j.1532-950X.1998.tb00150.x 9749511

[B78] Kurzman ID MacEwen EG Rosenthal RC Adjuvant therapy for osteosarcoma in dogs: results of randomized clinical trials using combined liposome-encapsulated muramyl tripeptide and cisplatin Clin Cancer Res 1995 Dec 1 12 1595 601 9815961

[B79] LaRue SM Withrow SJ Powers BE Limb-sparing treatment for osteosarcoma in dogs J Am Vet Med Assoc 1989 Dec 15 195 12 1734 44 2599960

[B80] Lascelles BD Dernell WS Correa MT Improved survival associated with postoperative wound infection in dogs treated with limb-salvage surgery for osteosarcoma Ann Surg Oncol 2005 Dec 12 12 1073 83 10.1245/ASO.2005.01.011 16252138

[B81] Liptak JM Dernell WS Straw RC Proximal radial and distal humeral osteosarcoma in 12 dogs J Am Anim Hosp Assoc 2004 Nov-Dec 40 6 461 7 15533966 10.5326/0400461

[B82] Liptak JM Dernell WS Ehrhart N Cortical allograft and endoprosthesis for limb-sparing surgery in dogs with distal radial osteosarcoma: a prospective clinical comparison of two different limb-sparing techniques Vet Surg 2006 Aug 35 6 518 33 10.1111/j.1532-950X.2006.00185.x 16911152

[B83] MacEwen EG Kurzman ID Rosenthal RC Therapy for osteosarcoma in dogs with intravenous injection of liposome-encapsulated muramyl tripeptide J Natl Cancer Inst 1989 Jun 21 81 12 935 8 10.1093/jnci/81.12.935 2733037

[B84] Mauldin GN Matus RE Withrow SJ Patnaik AK Canine osteosarcoma. Treatment by amputation versus amputation and adjuvant chemotherapy using doxorubicin and cisplatin J Vet Intern Med 1988 Oct-Dec 2 4 177 80 10.1111/j.1939-1676.1988.tb00313.x 3230557

[B85] McMahon M Mathie T Stingle N Adjuvant carboplatin and gemcitabine combination chemotherapy postamputation in canine appendicular osteosarcoma J Vet Intern Med 2011 May 25 3 511 7 10.1111/j.1939-1676.2011.0697.x 21488959 PMC3807042

[B86] McNeill CJ Overlev B Shofer FS Characterization of the biological behaviour of appendicular osteosarcoma in Rottweilers and a comparison with other breeds: a review of 258 dogs Vet Comp Oncol 2007 Jun 5 2 90 8 10.1111/j.1476-5829.2006.00116.x 19754792

[B87] Mehl ML Seguin B Dernell WS Survival analysis of one versus two treatments of local delivery cisplatin in a biodegradable polymer for canine osteosarcoma Vet Comp Oncol 2005 Jun 3 2 81 6 10.1111/j.1476-5810.2005.00072.x 19379216

[B88] Meyer JA Dueland RT MacEwen EG Canine osteogenic sarcoma treated by amputation and MER: an adverse effect of splenectomy on survival Cancer 1982 Apr 15 49 8 1613 6 10.1002/1097-0142(19820415)49:8<1613::AID-CNCR2820490814>3.0.CO;2-R 6950801

[B89] Miller AG Morley PS Rao S Anemia is associated with decreased survival time in dogs with lymphoma J Vet Intern Med 2009 23 116 122 10.1111/j.1939-1676.2008.0210.x 19138381

[B90] Moore AS Dernell WS Ogilvie GK Doxorubicin and BAY 12-9566 for the treatment of osteosarcoma in dogs: a randomized, double-blind, placebo-controlled study J Vet Intern Med 2007 Jul-Aug 21 4 783 90 10.1111/j.1939-1676.2007.tb03022.x 17708400

[B91] Mullins MN Lana SE Dernell WS Cyclooxygenase-2 expression in canine appendicular osteosarcomas J Vet Intern Med 2004 Nov-Dec 18 6 859 65 10.1111/j.1939-1676.2004.tb02633.x 15638270

[B92] Petty JC Lana SE Thamm DH Glucose transporter 1 expression in canine osteosarcoma Vet Comp Oncol 2008 Jun 6 2 133 40 10.1111/j.1476-5829.2007.00155.x 19178673

[B93] Phillips B Powers BE Dernell WS Use of single-agent carboplatin as adjuvant or neoadjuvant therapy in conjunction with amputation for appendicular osteosarcoma in dogs J Am Anim Hosp Assoc 2009 Jan-Feb 45 1 33 8 19122062 10.5326/0450033

[B94] Powers BE Withrow SJ Thrall DE Percent tumor necrosis as a predictor of treatment response in canine osteosarcoma Cancer 1991 Jan 1 67 1 126 34 10.1002/1097-0142(19910101)67:1<126::AID-CNCR2820670123>3.0.CO;2-7 1985708

[B95] Saam DE Liptak JM Stalker MJ Chun R Predictors of outcome in dogs treated with adjuvant carboplatin for appendicular osteosarcoma: 65 cases (1996-2006) J Am Vet Med Assoc 2011 Jan 15 238 2 195 206 10.2460/javma.238.2.195 21235373

[B96] Selvarajah GT Kirpensteijn J van Wolferen ME Gene expression profiling of canine osteosarcoma reveals genes associated with short and long survival times Mol Cancer 2009 Sep 7 8 72 10.1186/1476-4598-8-72 19735553 PMC2746177

[B97] Shapiro W Fossum TW Kitchell BE Use of cisplatin for treatment of appendicular osteosarcoma in dogs J Am Vet Med Assoc 1988 Feb 15 192 4 507 11 3163684

[B98] Sharili AS Allen S Smith K Expression of Snail2 in long bone osteosarcomas correlates with tumour malignancy Tumour Biol 2011 Jan 5 32 3 515 526 10.1007/s13277-010-0146-1 21207222 PMC3109975

[B99] Sottnik JL Rao S Lafferty MH Association of blood monocyte and lymphocyte count and disease-free interval in dogs with osteosarcoma J Vet Intern Med 2010 Nov-Dec 24 6 1439 44 10.1111/j.1939-1676.2010.0591.x 20840314

[B100] Spodnick GJ Berg J Rand WM Prognosis for dogs with appendicular osteosarcoma treated by amputation alone: 162 cases (1978-1988) J Am Vet Med Assoc 1992 Apr 1 200 7 995 9 1577656

[B101] Stein TJ Holmes KE Muthuswamy A Characterization of β-catenin expression in canine osteosarcoma Vet Comp Oncol 2011 Mar 9 1 65 73 10.1111/j.1476-5829.2010.00236.x 21303455 PMC3099435

[B102] Straw RC Withrow SJ Richter SL Amputation and cisplatin for treatment of canine osteosarcoma J Vet Intern Med 1991 Jul-Aug 5 4 205 10 10.1111/j.1939-1676.1991.tb00950.x 1941754

[B103] Thamm DH O’Brien MG Vail DM Serum vascular endothelial growth factor concentrations and postsurgical outcome in dogs with osteosarcoma Vet Comp Oncol 2008 Jun 6 2 126 32 10.1111/j.1476-5829.2007.00153.x 19178672

[B104] Thompson JP Fugent MJ Evaluation of survival times after limb amputation, with and without subsequent administration of cisplatin, for treatment of appendicular osteosarcoma in dogs: 30 cases (1979-1990) J Am Vet Med Assoc 1992 Feb 15 200 4 531 3 1559895

[B105] Vail DM Kurzman ID Glawe PC STEALTH liposome-encapsulated cisplatin (SPI-77) versus carboplatin as adjuvant therapy for spontaneously arising osteosarcoma (OSA) in the dog: a randomized multicenter clinical trial Cancer Chemother Pharmacol 2002 Aug 50 2 131 6 10.1007/s00280-002-0469-8 12172978

[B106] Withrow SJ Thrall DE Straw RC Intra-arterial cisplatin with or without radiation in limb-sparing for canine osteosarcoma Cancer 1993 Apr 15 71 8 2484 90 10.1002/1097-0142(19930415)71:8<2484::AID-CNCR2820710810>3.0.CO;2-D 8453572

[B107] Withrow SJ Liptak JM Straw RC Biodegradable cisplatin polymer in limb-sparing surgery for canine osteosarcoma Ann Surg Oncol 2004 Jul 11 7 705 13 10.1245/ASO.2004.10.008 15231525

