# Supplementary material for: Prognostic factors in canine appendicular osteosarcoma – a meta-analysis
Source: BMC Vet Res. 2012 May 15;8:56. doi: 10.1186/1746-6148-8-56 (PMC3482154; doi:10.1186/1746-6148-8-56)
Supplement: Additional file 1 — Papers (55) that met the criteria for inclusion in this study [57-107]. Table S1. Studies selected for meta-analysis. [file 1746-6148-8-56-S1.docx]

Additional file I: Papers (55) that met the criteria for inclusion in this study

Bacon NJ, Erhart NP, Dernell WS, et al.: Use of alternating administration of carboplatin and doxorubicin in dogs with microscopic metastases after amputation for appendicular osteosarcoma: 50 cases (1999-2006). [J Am Vet Med Assoc.](javascript:AL_get(this,%20'jour',%20'J%20Am%20Vet%20Med%20Assoc.');) 2008 May 15;232(10):1504-10.

Bailey D, Erb H, Williams L, et al.: Carboplatin and doxorubicin combination chemotherapy for the treatment of appendicular osteosarcoma in the dog. J Vet Intern Med. 2003;17:199–205.

Bech-Nielsen S, Brodey RS, Fidler IJ, et al.: The effect of BCG on in vitro immune reactivity and clinical course in dogs treated surgically for osteosarcoma. [Eur J Cancer.](javascript:AL_get(this,%20'jour',%20'Eur%20J%20Cancer.');) 1977 Jan;13(1):33-41.

Berg J, Weinstein MJ, Schelling SH, Rand WM: Treatment of dogs with osteosarcoma by administration of cisplatin after amputation or limb-sparing surgery: 22 cases (1987-1990). [J Am Vet Med Assoc.](javascript:AL_get(this,%20'jour',%20'J%20Am%20Vet%20Med%20Assoc.');) 1992 Jun 15;200(12):2005-8.

Berg J, Weinstein MJ, Springfield DS, Rand WM: Results of surgery and doxorubicin chemotherapy in dogs with osteosarcoma. [J Am Vet Med Assoc.](javascript:AL_get(this,%20'jour',%20'J%20Am%20Vet%20Med%20Assoc.');) 1995 May 15;206(10):1555-60.

Berg J, Gebhardt MC, Rand WM: Effect of timing of postoperative chemotherapy on survival of dogs with osteosarcoma. [Cancer.](javascript:AL_get(this,%20'jour',%20'Cancer.');) 1997 Apr 1;79(7):1343-50.

Bergman PJ, MacEwen EG, Kurzman ID, et al.: Amputation and carboplatin for treatment of dogs with osteosarcoma: 48 cases (1991 to 1993). [J Vet Intern Med.](javascript:AL_get(this,%20'jour',%20'J%20Vet%20Intern%20Med.');) 1996 Mar-Apr;10(2):76-81.

Biller BJ, Guth A, Burton JH, Dow SW: Decreased ratio of CD81 T cells to regulatory T cells associated with decreased survival in dogs with osteosarcoma. J Vet Intern Med. 2010;24:1118–1123.

Chun R, Kurzman ID, Couto CG, et al.: Cisplatin and doxorubicin combination chemotherapy for the treatment of canine osteosarcoma: a pilot study. J Vet Intern Med. 2000;14:495–498.

Chun R, Garrett LD, Henry C, et al.: Toxicity and efficacy of cisplatin and doxorubicin combination chemotherapy for the treatment of canine osteosarcoma. [J Am Anim Hosp Assoc.](javascript:AL_get(this,%20'jour',%20'J%20Am%20Anim%20Hosp%20Assoc.');) 2005 Nov-Dec;41(6):382-7.

DiResta GR, Aiken SW, Brown HK, et al.: Use of an artificial lymphatic system during carboplatin infusion to improve canine osteosarcoma blood flow and clinical response. Ann Surg Oncol. 2007 Aug;14(8):2411-21.

Ehrhart N, Dernell WS, Hoffmann WE, et al.: Prognostic importance of alkaline phosphatase activity in serum from dogs with appendicular osteosarcoma: 75 cases (1990-1996). [J Am Vet Med Assoc.](javascript:AL_get(this,%20'jour',%20'J%20Am%20Vet%20Med%20Assoc.');) 1998 Oct 1;213(7): 1002-6.

Fieten H, Spee B, Ijzer J, et al.: Expression of hepatocyte growth factor and the proto-oncogenic receptor c-Met in canine osteosarcoma. Vet Pathol. 2009 Sep; 46(5):869-77.

Garzotto CK, Berg J, Hoffmann WE, Rand WM: Prognostic significance of serum alkaline phosphatase activity in canine appendicular osteosarcoma. J Vet Intern Med. 2000;14:587–592.

Hahn KA, Legendre AM, Talbott JR: The frequency of micronuclei in lymphocytes of dogs with osteosarcoma: a predictive variable for tumor response during cisplatin chemotherapy. [Cancer Epidemiol Biomarkers Prev.](javascript:AL_get(this,%20'jour',%20'Cancer%20Epidemiol%20Biomarkers%20Prev.');) 1996 Aug;5(8):653-6.

Hahn KA, Legendre AM, Schuller HM: Amputation and dexniguldipine as treatment for canine appendicular osteosarcoma. [J Cancer Res Clin Oncol.](javascript:AL_get(this,%20'jour',%20'J%20Cancer%20Res%20Clin%20Oncol.');) 1997;123(1):34-8.

Hillers KR, Dernell WS, Lafferty MH, et al.: Incidence and prognostic importance of lymph node metastases in dogs with appendicular osteosarcoma: 228 cases (1986-2003). J Am Vet Med Assoc. 2005 Apr 15;226(8): 1364-7.

Kent MS, Strom A, London CA, et al.: Alternating carboplatin and doxorubicin as adjunctive chemotherapy to amputation or limb-sparing surgery in the treatment of appendicular osteosarcoma in dogs. J Vet Intern Med. 2004;18:540–544.

Khanna C, Prehn J, Hayden D, et al.: A randomized controlled trial of octreotide pamoate long-acting release and carboplatin versus carboplatin alone in dogs with naturally occurring osteosarcoma: evaluation of insulin-like growth factor suppression and chemotherapy. [Clin Cancer Res.](javascript:AL_get(this,%20'jour',%20'Clin%20Cancer%20Res.');) 2002 Jul;8(7):2406-12.

Khanna C, Wan X, Bose S, et al.: The membrane-cytoskeleton linker ezrin is necessary for osteosarcoma metastasis. [Nat Med.](javascript:AL_get(this,%20'jour',%20'Nat%20Med.');) 2004 Feb;10(2):182-6.

Kirpensteijn J, Kik M, Rutteman GR, Teske E: Prognostic significance of a new histologic grading system for canine osteosarcoma. [Vet Pathol.](javascript:AL_get(this,%20'jour',%20'Vet%20Pathol.');) 2002 Mar;39(2):240-6.

Kirpensteijn J, Timmermans-Spran EP, van Garderen E, et al.: Growth hormone gene expression in canine normal growth plates and spontaneous osteosarcoma. [Mol Cell Endocrinol.](javascript:AL_get(this,%20'jour',%20'Mol%20Cell%20Endocrinol.');) 2002 Nov 29;197(1-2):179-85.

Kirpensteijn J, Kik M, Teske E, Rutteman GR: TP53 gene mutations in canine osteosarcoma. [Vet Surg.](javascript:AL_get(this,%20'jour',%20'Vet%20Surg.');) 2008 Jul;37(5):454-60.

Kow K, Thamm DH, Terry J, et al.: Impact of telomerase status on canine osteosarcoma patients. J Vet Intern Med. 2008 Nov-Dec;22(6):1366-72.

Kuntz CA, Asselin TL, Dernell WS, et al.: Limb salvage surgery for osteosarcoma of the proximal humerus: outcome in 17 dogs. [Vet Surg.](javascript:AL_get(this,%20'jour',%20'Vet%20Surg.');) 1998 Sep-Oct;27(5):417-22.

Kurzman ID, MacEwen EG, Rosenthal RC, et al.: Adjuvant therapy for osteosarcoma in dogs: results of randomized clinical trials using combined liposome-encapsulated muramyl tripeptide and cisplatin. [Clin Cancer Res.](javascript:AL_get(this,%20'jour',%20'Clin%20Cancer%20Res.');) 1995 Dec;1(12):1595-601.

LaRue SM, Withrow SJ, Powers BE, et al.: Limb-sparing treatment for osteosarcoma in dogs. [J Am Vet Med Assoc.](javascript:AL_get(this,%20'jour',%20'J%20Am%20Vet%20Med%20Assoc.');) 1989 Dec 15;195(12):1734-44.

Lascelles BD, Dernell WS, Correa MT, et al.: Improved survival associated with postoperative wound infection in dogs treated with limb-salvage surgery for osteosarcoma. Ann Surg Oncol. 2005 Dec;12(12):1073-83.

Liptak JM, Dernell WS, Straw RC, et al.: Proximal radial and distal humeral osteosarcoma in 12 dogs. J Am Anim Hosp Assoc. 2004 Nov-Dec;40(6):461-7.

Liptak JM, Dernell WS, Ehrhart N, et al.: Cortical allograft and endoprosthesis for limb-sparing surgery in dogs with distal radial osteosarcoma: a prospective clinical comparison of two different limb-sparing techniques. Vet Surg. 2006 Aug;35(6):518-33.

MacEwen EG, Kurzman ID, Rosenthal RC, et al.: Therapy for osteosarcoma in dogs with intravenous injection of liposome-encapsulated muramyl tripeptide. [J Natl Cancer Inst.](javascript:AL_get(this,%20'jour',%20'J%20Natl%20Cancer%20Inst.');) 1989 Jun 21;81(12): 935-8.

Mauldin GN, Matus RE, Withrow SJ, Patnaik AK: Canine osteosarcoma. Treatment by amputation versus amputation and adjuvant chemotherapy using doxorubicin and cisplatin. [J Vet Intern Med.](javascript:AL_get(this,%20'jour',%20'J%20Vet%20Intern%20Med.');) 1988 Oct-Dec; 2(4):177-80.

McMahon M, Mathie T, Stingle N, et al.: Adjuvant carboplatin and gemcitabine combination chemotherapy postamputation in canine appendicular osteosarcoma. J Vet Intern Med. 2011 May; 25(3):511-7.

McNeill CJ, Overlev B, Shofer FS, et al.: Characterization of the biological behaviour of appendicular osteosarcoma in Rottweilers and a comparison with other breeds: a review of 258 dogs. Vet Comp Oncol. 2007 Jun;5(2):90-8.

Mehl ML, Seguin B, Dernell WS, et al.: Survival analysis of one versus two treatments of local delivery cisplatin in a biodegradable polymer for canine osteosarcoma. Vet Comp Oncol. 2005 Jun; 3(2):81-6.

Meyer JA, Dueland RT, MacEwen EG, et al.: Canine osteogenic sarcoma treated by amputation and MER: an adverse effect of splenectomy on survival. [Cancer.](javascript:AL_get(this,%20'jour',%20'Cancer.');) 1982 Apr 15;49(8):1613-6.

Miller AG, Morley PS, Rao S, et al.: Anemia is associated with decreased survival time in dogs with lymphoma. J Vet Intern Med. 2009;23:116–122.

Moore AS, Dernell WS, Ogilvie GK, et al.: Doxorubicin and BAY 12-9566 for the treatment of osteosarcoma in dogs: a randomized, double-blind, placebo-controlled study. J Vet Intern Med. 2007 Jul-Aug;21(4):783-90.

Mullins MN, Lana SE, Dernell WS, et al.: Cyclooxygenase-2 expression in canine appendicular osteosarcomas. J Vet Intern Med. 2004 Nov-Dec;18(6):859-65.

Petty JC, Lana SE, Thamm DH, et al.: Glucose transporter 1 expression in canine osteosarcoma. Vet Comp Oncol. 2008 Jun;6(2):133-40.

Phillips B, Powers BE, Dernell WS, et al.: Use of single-agent carboplatin as adjuvant or neoadjuvant therapy in conjunction with amputation for appendicular osteosarcoma in dogs. [J Am Anim Hosp Assoc.](javascript:AL_get(this,%20'jour',%20'J%20Am%20Anim%20Hosp%20Assoc.');) 2009 Jan-Feb;45(1):33-8.

Powers BE, Withrow SJ, Thrall DE, et al.: Percent tumor necrosis as a predictor of treatment response in canine osteosarcoma. [Cancer.](javascript:AL_get(this,%20'jour',%20'Cancer.');) 1991 Jan 1;67(1):126-34.

Saam DE, Liptak JM, Stalker MJ, Chun R: Predictors of outcome in dogs treated with adjuvant carboplatin for appendicular osteosarcoma: 65 cases (1996-2006). J Am Vet Med Assoc. 2011 Jan 15;238(2):195-206.

Selvarajah GT, Kirpensteijn J, van Wolferen ME, et al.: Gene expression profiling of canine osteosarcoma reveals genes associated with short and long survival times. Mol Cancer. 2009 Sep 7; 8:72.

Shapiro W, Fossum TW, Kitchell BE, et al.: Use of cisplatin for treatment of appendicular osteosarcoma in dogs. [J Am Vet Med Assoc.](javascript:AL_get(this,%20'jour',%20'J%20Am%20Vet%20Med%20Assoc.');) 1988 Feb 15;192(4):507-11.

Sharili AS, Allen S, Smith K, et al.: Expression of Snail2 in long bone osteosarcomas correlates with tumour malignancy. [Tumour Biol.](javascript:AL_get(this,%20'jour',%20'Tumour%20Biol.');) 2011 Jan 5.

Sottnik JL, Rao S, Lafferty MH, et al.: Association of blood monocyte and lymphocyte count and disease-free interval in dogs with osteosarcoma. J Vet Intern Med. 2010 Nov-Dec;24(6):1439-44.

Spodnick GJ, Berg J, Rand WM, et al.: Prognosis for dogs with appendicular osteosarcoma treated by amputation alone: 162 cases (1978-1988). [J Am Vet Med Assoc.](javascript:AL_get(this,%20'jour',%20'J%20Am%20Vet%20Med%20Assoc.');) 1992 Apr 1;200(7):995-9.

Stein TJ, Holmes KE, Muthuswamy A, et al.: Characterization of β-catenin expression in canine osteosarcoma. [Vet Comp Oncol.](javascript:AL_get(this,%20'jour',%20'Vet%20Comp%20Oncol.');) 2011 Mar;9(1):65-73.

Straw RC, Withrow SJ, Richter SL, et al.: Amputation and cisplatin for treatment of canine osteosarcoma. [J Vet Intern Med.](javascript:AL_get(this,%20'jour',%20'J%20Vet%20Intern%20Med.');) 1991 Jul-Aug;5(4):205-10.

Thamm DH, O’Brien MG, Vail DM: Serum vascular endothelial growth factor concentrations and postsurgical outcome in dogs with osteosarcoma. Vet Comp Oncol. 2008 Jun;6(2):126-32.

Thompson JP, Fugent MJ: Evaluation of survival times after limb amputation, with and without subsequent administration of cisplatin, for treatment of appendicular osteosarcoma in dogs: 30 cases (1979-1990). [J Am Vet Med Assoc.](javascript:AL_get(this,%20'jour',%20'J%20Am%20Vet%20Med%20Assoc.');) 1992 Feb 15;200(4):531-3.

Vail DM, Kurzman ID, Glawe PC, et al.: STEALTH liposome-encapsulated cisplatin (SPI-77) versus carboplatin as adjuvant therapy for spontaneously arising osteosarcoma (OSA) in the dog: a randomized multicenter clinical trial.[Cancer Chemother Pharmacol.](javascript:AL_get(this,%20'jour',%20'Cancer%20Chemother%20Pharmacol.');) 2002 Aug;50(2):131-6.

Withrow SJ, Thrall DE, Straw RC, et al.: Intra-arterial cisplatin with or without radiation in limb-sparing for canine osteosarcoma. [Cancer.](javascript:AL_get(this,%20'jour',%20'Cancer.');) 1993 Apr 15;71(8):2484-90.

Withrow SJ, Liptak JM, Straw RC, et al.: Biodegradable cisplatin polymer in limb-sparing surgery for canine osteosarcoma. Ann Surg Oncol. 2004 Jul;11(7):705-13.

Additional file II: Studies selected for meta-analysis

| Study (year) | Elevated SALP | | | | (prox.) humerus | | | | Age at diagnosis | | | | | |
| --- | --- | --- | --- | --- | --- | --- | --- | --- | --- | --- | --- | --- | --- | --- |
|  | ST | | DFI | | ST | | DFI | | ST | | | DFI | | |
|  | HR | MST | HR | MDFI | HR | MST | HR | MDFI | HR | MST | p | HR | MDFI | p |
| McMahon, 2011 |  |  |  |  |  |  |  | • |  |  |  |  |  |  |
| Saam, 2011 | • |  |  |  | • | • |  |  | • | • |  |  |  |  |
| Sottnik, 2010 |  |  | • | • |  |  | • | • |  |  |  | • | • |  |
| Selvarajah, 2009 | • |  | • |  |  |  |  |  | • |  |  |  |  |  |
| Phillips, 2009 | • | • | • | • | • | • | • | • |  | • |  | • | • |  |
| Miller, 2009 |  |  |  |  |  |  |  |  | • |  |  | • |  |  |
| Thamm, 2008 | • |  | • | • |  |  |  |  |  |  |  |  |  |  |
| Kow, 2008 |  | • | • | • |  | • |  | • |  |  |  |  |  |  |
| Moore, 2007 |  | • |  |  |  |  |  |  |  |  | • |  |  |  |
| McNeill, 2007 |  | • |  | • |  |  |  |  |  |  |  |  |  |  |
| Liptak, 2006 |  |  |  |  |  |  |  |  | • |  | • |  |  |  |
| Chun, 2005 |  | • |  | • |  |  |  |  |  |  |  |  |  |  |
| Hillers, 2005 |  |  |  | • |  |  |  | • |  |  |  |  |  |  |
| Kent, 2004 |  |  |  |  |  |  |  |  |  |  | • |  |  | • |
| Kirpensteijn,2002 | • |  | • |  |  |  |  |  |  |  |  |  |  |  |
| Vail, 2002 |  | • |  | • |  |  |  |  |  |  |  |  |  |  |
| Garzotto, 2000 | • | • |  |  |  |  |  |  |  |  |  |  |  |  |
| Bergman, 1996 |  |  |  |  | • |  | • |  |  |  |  |  |  |  |
| Spodnick, 1992 |  |  |  |  |  |  |  |  |  |  | • |  |  |  |
